# Ligament reconstruction for distal radioulnar joint instability with the biomechanical analysis: A case report

**DOI:** 10.1097/MD.0000000000040057

**Published:** 2024-10-11

**Authors:** Baiyang Zhang, Xilin Liu, Hongbin Sun

**Affiliations:** a Department of Hand and Foot Surgery, China-Japan Union Hospital, Jilin University, Jilin, China.

**Keywords:** distal radioulnar joint ligament reconstruction, finite element analysis, joint instability, triangular fibrocartilage complex

## Abstract

**Rationale:**

The aim of the study is to evaluate the clinical effects and feasibility on chronic distal radioulnar unstable joint (DRUJ) under wrist arthroscope triangular fibrocartilage complex (TFCC) repair and reconstruction. The biomechanical characteristics of the recovery process after treatment were analyzed using finite element modeling.

**Patient concerns:**

A patient with chronic DRUJ instability was treated with reconstruction of the distal radioulnar ligament using the Adams Berger method. Piano sign and forearm rotation tests were used to evaluate the function of the DRUJ. Grip power, range of motion, and visual analog scale scores were recorded at the last follow-up. Joint function was evaluated by the disability of the arm, shoulder, and hand score. A geometric model for the surgical repair of TFCC damage, meshing in finite element simulation, and stress distribution of the repaired ligament during forearm rotation were analyzed by finite element simulation.

**Diagnoses:**

The diagnosis was confirmed as chronic DRUJ instability.

**Interventions and outcomes:**

The patient had no postoperative complications and was followed-up for 6 months. Stability was achieved in all patients. The results of the stress and forearm rotation tests are negative. At the last follow-up, the grip power, disability of the arm, shoulder, and hand score, visual analog scale score, and range of motion of the wrist were significantly improved compared with the preoperative values (*P* < .05).

**Lessons:**

TFCC repair and reconstruction under wrist arthroscopy can effectively treat chronic DRUJ and improve wrist function. Our study established a three-dimensional finite element model of the entire DRUJ, which provided a digital visual platform for simulating the biomechanical features of the TFCC, DRUJ, and other structures in different states. This study demonstrated that the rotation angle of the wrist joint should be confined within 75° for a short period after surgery to avoid tearing the tendon as a result of excessive force. It also provides an intuitive simulation tool for the design of TFCC repair and the evaluation of curative effects.

## 1. Introduction

Ulnar styloid fracture is a common complication of wrist fracture and dislocation, contributing to 40% to 65% of distal fractures of the radius.^[1]^ There is still a clinical debate regarding the effects of ulnar styloid fractures on the incidence of distal radioulnar joint instability. May et al reported that ulnar styloid base fractures and significant ulnar styloid fractures with displacement increase the incidence of distal radioulnar joint instability.^[2]^ The anatomical structure of a stable distal radioulnar joint (DRUJ) includes components both inside and outside the articular capsule. The pronator quadratus, Interosseous Membrane, sixth dorsal compartment, and tendinous sheath of the extensor carpi ulnaris; the in-capsule structures comprising the osseous articular surface, triangular fibrocartilage complex (TFCC), and articular capsule are among the extra-capsule structures.^[3,4]^ Additionally, because of their deep anatomical placement, extra-capsule structures are not readily exposed to damage. The displacement of distal fracture fragments in distal fractures of the radius tends to cause TFCC damage of varying degrees for in-capsule structures, while TFCC damage is primarily responsible for DRUJ stability.^[5,6]^

The TFCC is a crucial component of the ulnar side of the wrist joint and plays an important role in maintaining the stability of the DRUJ. TFCC damage is a common cause of discomfort on the ulnar side of the wrist joint, limited activity, poor grip, and DRUJ instability. If diagnosis and treatment are not performed promptly, TFCC injuries will significantly interfere with the patient’s employment and life.^[7,8]^

TFCC injuries are one of the most common causes of wrist joint instability. Early confirmation of the damaging nature and precise fracture location can be crucial for guiding the selection of clinical treatment methods.^[9,10]^ Ulnar-sided wrist pain is frequently caused by damage to the TFCC. Patients with persistent symptoms, despite conservative treatment, should seek surgery. Recently, advances in wrist joint arthroscopy have enabled accurate diagnosis of TFCC injury and minimally invasive.^[11]^ The major blood supply to the TFCC includes the branches of the ulnar artery, dorsal interosseous artery, radial artery, and palmar and dorsal branches of the anterior interosseous artery. Except for the radial edge, the peripheral 20% of the TFCC had abundant vessels, whereas its central region (80%) had no vessels. Hence, if tissues with a blood supply are damaged, repair is required for recovery.^[12–14]^ In open reduction and internal fixation, it is challenging to accurately align the articular bone mass, and it tends to ignore damage to soft tissues such as the TFCC and ligament, resulting in wrist pain and limited activity.

Currently, there are no universal standards for the treatment of TFCC damage,^[15]^ and the most frequently adopted conservative approach is ineffective.^[16]^ The arthroscope has played an increasingly important role in the diagnosis and treatment of TFCC damage as a result of advances in minimally invasive treatment.^[17,18]^ The DRUJ is located at the distal end of the forearm, and the transfer of wrist force load, forearm pronation, and forearm supination depends on its structural integrity.^[19]^ The understanding of distal anatomy and radius fractures, as well as the biomechanics of wrist joints, has been continuously developed with research. Thus, the concept and methods for the treatment of TFCC damage have been continuously improved. The DRUJ is a crucial component of normal function of the wrist joint and forearm. Approximately 8% of fractures and dislocations of the forearm were caused by DRUJ dislocation. Clinical treatment and effective rehabilitation require a deep understanding of the biomechanical mechanism of DRUJ fractures owing to their vulnerability to damage.^[20–22]^ Since 1988, when wrist joint arthroscopy was first used to treat TFCC damage, it has gradually become the primary surgical method.^[23]^ The clinical efficacy of wrist arthroscopy in the treatment of triangular fibrocartilage complex Palmer 1B injury was evaluated in a retrospective study, and a finite element model was used to analyze the precautions in the recovery process after treatment. In this study, TFCC repair was used to treat stage I distal radius fractures coupled with acute DRUJ instability during wrist joint arthroscopy. Through 6 months follow-up, the clinical efficacy of stage I TFCC repair for acute DRUJ after distal fracture of the radius was evaluated, and a finite element simulation was used to analyze the stress distribution of the repaired ligament during forearm rotation.

## 2. Case report

The patient was a 19-year-old male, who was admitted to the hospital after experiencing wrist pain and limited function for 3 months due to wrist trauma. Preoperative examination revealed a toothache in the ulnar nasopharynx fossa and positive push and pressure tests. Dorsal dislocation of the ulnar head could have resulted from forearm pronation. While MRI revealed TFCC signal intensity and morphological changes, radiography showed no abnormalities.

**Figure 1.**
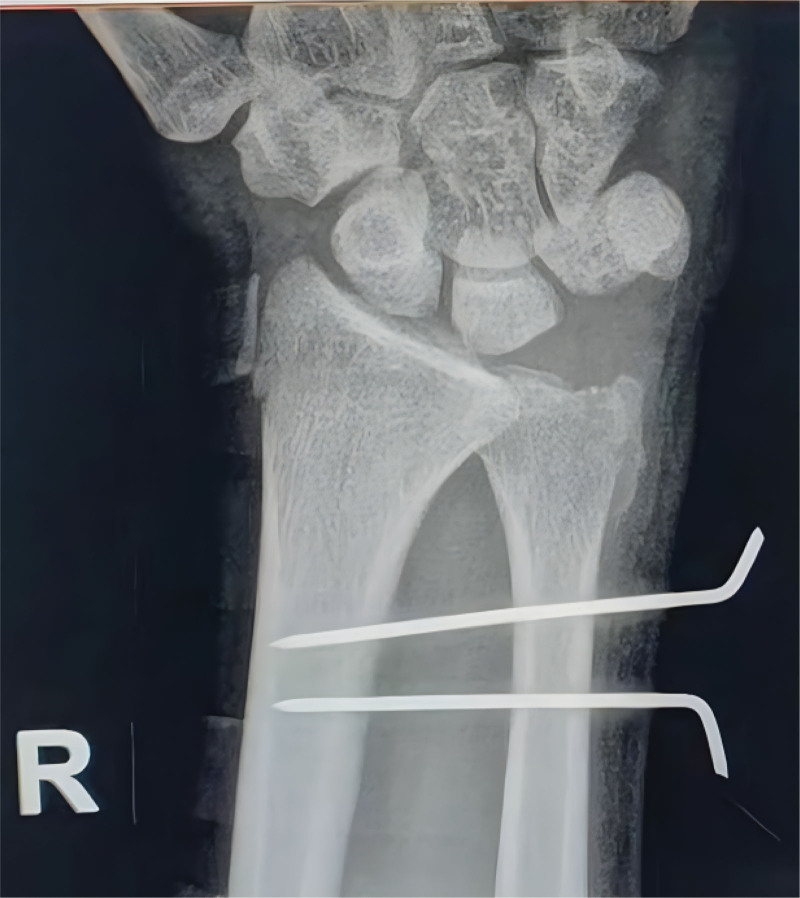
Posteroanterior radiograph after the initial surgery.

The pneumatic tourniquet was applied to the upper arm of the patient in a supine position. The affected limb was then placed flat on the operating table with elbow joint flexion at 90° and shoulder joint abduction at 90°. After fixation, traction of 10 to 15 pounds was applied to the affected limb by using a traction frame. First, a 3/4 approach was established. A number 10 needle was used to puncture the radial wrist joint, approximately 0.5 cm from the distal Lister node at the distal radius. The wrist joint cavity was injected with 5 to 15 mL normal saline. After the joint cavity was properly filled, the skin was cut longitudinally with a sharp blade. The soft tissues were then bluntly separated using straight-tissue forceps to expose the articular capsule. The 3 to 4, 6R, midcarpal radial, and midcarpal ulnar portals were introduced as standard arthroscopic portals. Fibrous scar tissue of the wrist joint cavity and hypertrophic synovial tissues were excised with a planer under a wrist joint arthroscope to expose the palmar DRUJ. The oblique osseous canal was drilled from the radial side of the ulnar styloid process to the ulnar neck, and the anterior and posterior osseous canals were drilled on the ulnar side of the radius. The free palmaris longus tendon was removed from the radial bony canal, and the volar and dorsal DRUJ were bypassed. It passes through the ulnar osseous canal and then wounds around 1 cycle from the neck of the ulna. To repair the dorsal carpal sheath, the DRUJ was reset to the neutral position of the wrist joint. The tendon was tightened, knotted, and repaired. Two Kirschner wires were used to repair the space between the radius and ulna (Fig. 1).

According to the disability of the arm, shoulder, and hand function score and visual analog scale score in the preoperative and last postoperative follow-up, the patient was evaluated by 3 doctors who did not participate in all his surgeries. The affected wrist joint range of motion was measured before and after the operation, as was the grip strength of the affected and healthy sides. After surgery, rest and exercise pain significantly improved. Both the forearm rotation test and the DRUJ piano sign test yielded negative results, and the grip strength, disability of the arm, shoulder, and hand score, visual analog scale score, wrist joint dorsiflexion, palmar flexion, pronation, and supination of the affected limb were significantly improved compared with those before the operation at the last follow-up (Table 1). No complications occurred during follow-up. This study was conducted in accordance with the Declaration of Helsinki guidelines. This study was approved by the Ethics Committee of the China-Japan Union Hospital of Jilin University. All participants provided written informed consent prior to participation.

**Table 1:** Comparison of the indexes at pre- and post-operation (n = 6, *x* ± *s*).

Time	Grip power (kg)	DASH score	VAS score	Range of motion (°)			
				Dorsiflexion	Volarflexion	Pronation	Supination
Preoperative	31.23 ± 1.34	21.42 ± 2.09	6.12 ± 1.07	62.73 ± 5.61	61.59 ± 6.74	68.98 ± 7.81	69.83 ± 9.12
Last follow-up	38.06 ± 1.56	7.38 ± 1.13	1.86 ± 0.31	68.24 ± 4.71	70.52 ± 5.03	77.24 ± 6.93	82.06 ± 5.85
Statistic	*t* = 1.82	*t* = 1.9	*t* = 1.92	*t* = 2.01	*t* = 2.12	*t* = 2.26	*t* = 1.86
	*P* = .045	*P* = .042	*P* = .04	*P* = .041	*P* = .039	*P* = .03	*P* = .043

DASH = disability of the arm, shoulder, and hand, VAS = visual analog scale.

### 2.1. Finite element simulation

**Figure 2.**
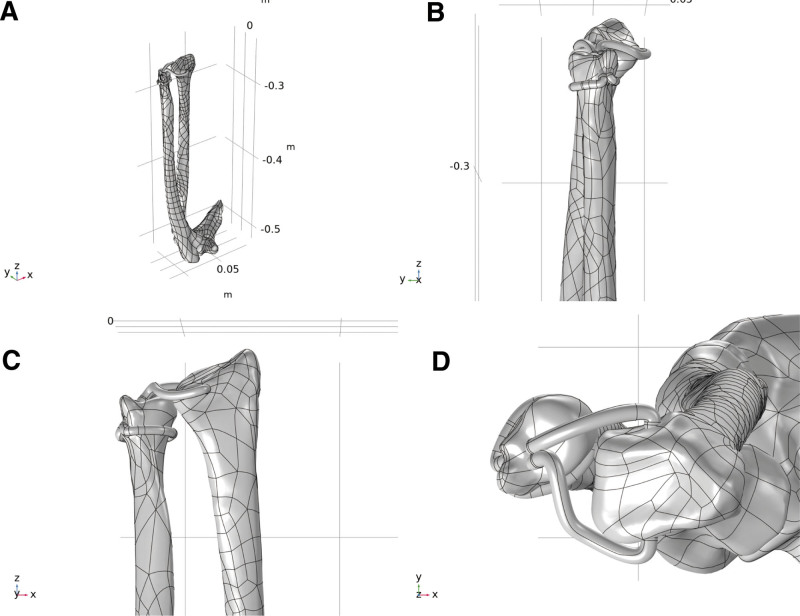
Geometric model for surgical repairment of TFCC damage. (A) Overall perspective. (B) Localized zoomed in view along the *X*-axis. (C) Localized zoomed in view along the *Y*-axis. (D) Localized zoomed in view along the *Z*-axis. TFCC = triangular fibrocartilage complex.

After the operation, a TFCC finite element simulation was performed to assess the effect of TFCC repair. To obtain slice scanning data of the ulna and radius (in DICOM format), the patient’s forearm was scanned using computed tomography (CT) (Optima, General Electric Company), with a scanning thickness of 3 mm. Gypsum and other materials were visible in the layer-scanning images, which comprised the CT data. Thus, for finite element simulations, it is necessary to extract the geometric information of the radius, ulna, and humerus and then convert them into geometric models that can be described by continuous functions. The 3D surface models of the ulna, radius, and elbow joint of the humerus were extracted from layer-scanning data using Mimics software (Materialise Co., Ltd, China) and then imported into Geomagic software (3D Systems Co., Ltd, China) to smooth the surfaces and establish 3D solid models. The smoothed 3D models were then imported into SolidWorks (Dassault Systems SolidWorks Co., Ltd, China), and geometric models of palmaris longus tendon in SolidWorks were constructed based on the surgical reconstruction method.^[24]^ Finally, the geometric models were imported into COMSOL (COMSOL Co., Ltd, China) as the geometric models of the finite element simulation (Fig. 2).

**Figure 3.**
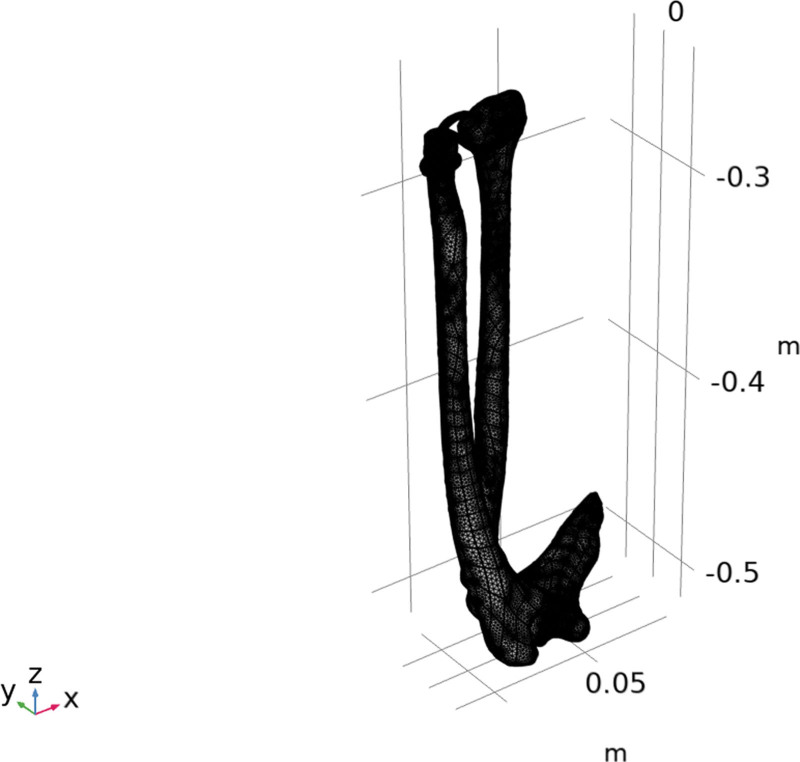
Meshing in finite element simulation.

The ulna, humerus, radius, and humerus were coupled by ball joints in the multibody dynamics model, upon which the model was built. The joints allowed rotational movement, but translational movement was restricted, with the joint centerline along the ulnar and radial axes. With an elastic modulus of 17.9 GPa, a Poisson ratio of 0.62, and a density of 2.0 kg/m^3^, the ulna, radius, and humerus were set as cortical bones.^[25]^ The elastic modulus of the tendon was 200 MPa, and the Poisson ratio was 0.4.^[26]^ Tetrahedral elements were also used for the model meshing, and 703 and 601 elements were generated, as shown in Figure 3.

In the simulation, the wrist joint was rotated 90° clockwise and counterclockwise from the middle position to evaluate the relationship between the wrist joint rotation angle and tendon force. A steady-state solver was used because the low rotation speed of the wrist joint allowed inertia to be ignored. The equation for force equilibrium is expressed as

$$\begin{array}{l} \nabla \cdot {\left(FS\right)}^{T}+f_{V}=0\;\;\; \end{array}$$ (1)

where *F* is the deformation gradient and F=I+∇u, **u** is the displacement vector, *S* is the second Piola–Kirchhoff stress, and **f**_*V*_ is the volumetric force.

## 3. Results

**Figure 4.**
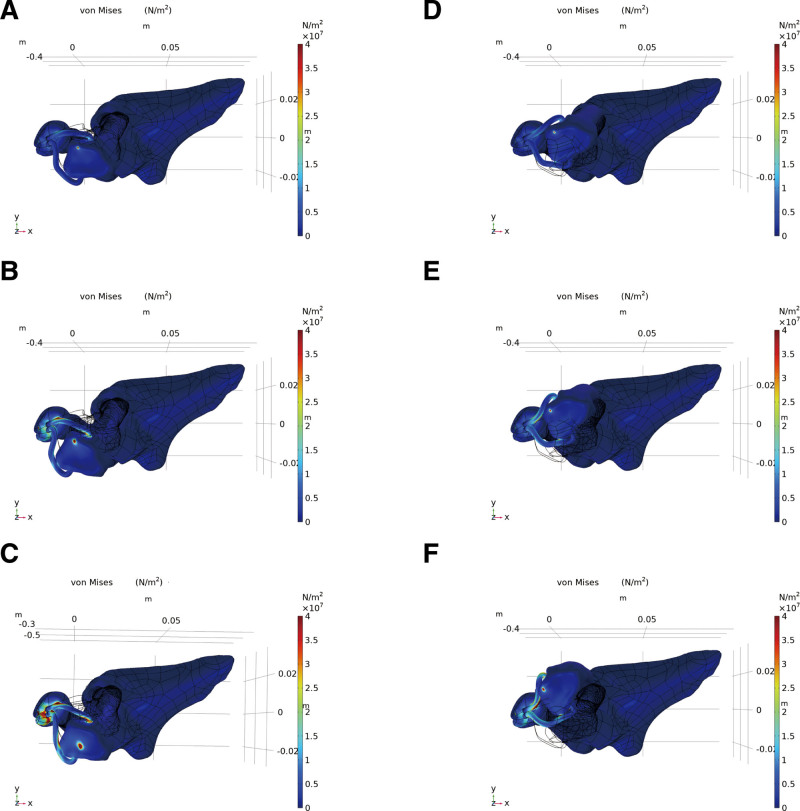
Stress distribution. (A) 18° clockwise. (B) 36° clockwise. (C) 54° clockwise. (D) 18° counterclockwise. (E) 36° counterclockwise. (F) 54° counterclockwise.

**Figure 5.**
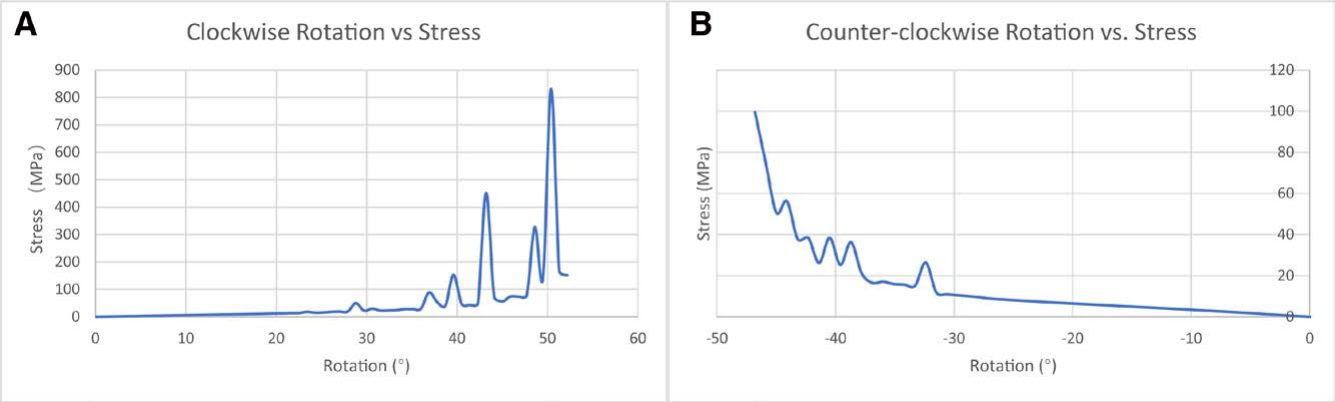
The relationship between rotation angle and average stress of tendon.

Figure 4 shows the von Mises effective stress distribution during clockwise and anticlockwise rotations of the wrist joint from the center. The joint between the tendon and ulna, which experienced the most stress, increased significantly as the rotation angle increased. We analyzed the relationship between the average force on the tendon and rotation angle to explore the relationship between the rotation angle and tendon force, as shown in Figure 5. The tendon force increased significantly when the rotation angle exceeded 35° clockwise or 30° anticlockwise. Therefore, it is recommended that the rotation angle of the wrist joint be confined within 75° for a short period after surgery to avoid tearing the tendon as a result of excessive force.

## 4. Discussion

The styloid process of the ulna, an important structural element of the ulnar side of the wrist joint stability, and the TFCC are closely related; therefore, a fracture of the styloid process of the ulna cannot be considered a simple isolated fracture clinically. Patients with DRUJ instability and TFCC damage must undergo surgical repair. Under direct visualization, internal fixation combined with wrist joint arthroscopy can accurately repair TFCC. Early functional exercise after surgery can prevent joint stiffness, although wrist joint arthroscopy is complex.^[27]^

The benefits of minimally invasive surgery are demonstrated by the arthroscopic treatment of ulnar styloid fractures, which can effectively reduce and fix the fracture favorably for fracture healing and also have an impact on damage to the TFCC and other structures in the wrist joint for early treatment. It applies to people who avoid chronic wrist pain or wrist joint instability and have significant separation of the ulnar styloid fracture mass, with TFCC damage and distal fracture of the radius.

Both digital and mathematical methods were applied for modeling using the finite element method. The known number of nodes, coordinate system of each node, material characteristics, and other conditions are then used to build an approximate solution for each unit. To quantify the actual complex problems, a total solution of this domain was deduced for quantitative analysis, which quantifies the actual complex issues.^[28–31]^ The application of the finite element analysis method in orthopedic research was first reported in 1972.^[32,33]^ Recent studies have focused on joint stress analysis, fracture risk prediction, and recommendations for internal fixation design and surgical strategy.^[34–37]^ The development of CT, MRI, and other imaging and scientific technologies has made continuous progress, providing accurate original data for orthopedic finite element research.

Based on finite element simulation, after TFCC reconstruction, the wrist joint rotation angle of the patient was recommended to be within 75° in the short term. Because the tendon is a biomaterial, it gradually stretches under an external force over time as a result of viscoelasticity. Therefore, the patient’s tendon stretched to a certain extent after the rehabilitation exercise, gradually increasing the range of wrist rotation. The CT image data from the participants were used to generate the general data in this study. After establishing the basic model, it was validated by comparing it with published experimental data. The selection of relevant boundary conditions and load cases was based on the general methods published in previous studies. Nevertheless, it is necessary to understand that finite element analysis is a mathematical and physical method for computer-based simulation calculation and analysis that has results close to the exact solution. Even with a high degree of precision, the model cannot be entirely consistent with the mechanical properties of the organisms. Our study established a three-dimensional finite element model of the entire DRUJ, which provided a digital visual platform for simulating the biomechanical features of the TFCC, DRUJ, and other structures in different states. It also provides an intuitive simulation tool for the design of TFCC repair and the evaluation of curative effects.

The idea that the TFCC is the internal structure needed to keep the DRUJ stable was initially proposed in 1981.^[8]^ However, there is still uncertainty regarding the physiological function of the distal radiometacarpal ligament^[38]^ proposed that TFCC deep fiber was the dominant internal stable structure of DRUJ,^[39]^ on the other hand, refuted the results. According to Hagert,^[40]^ the above 2 experiments were correct in 1994. Clinically, deep ligament damage is one of the causes of DRUJ instability. Some of the physical examinations include the stress test, piano sign test,^[41]^ press test,^[42]^ and ulnar fovea sign.^[43]^

DRUJ instability causes ulnar and radial TFCC tears, styloid process base fractures, and annular avulsion fractures as a result of ligament damage. The goal of the DRUJ ligament anatomical reconstruction is to achieve anatomical reconstruction of the ligament and accurate selection of anatomical positions. The starting and ending points of the osseous canal are necessary for a successful operation. Anatomical reconstruction can be accomplished with autologous palmaris longus tendon transplantation for ligament reconstruction with an ideal prognosis.^[44,45]^ Various TFCC repairs are currently performed clinically under arthroscopy. As the deep layer of the torn TFCC is not directly reattached to the ulnar fossa, some researchers believe that straightforward soft tissue suture technology (the torn TFCC was sutured to the tendinous sheath of the extensor carpi ulnaris and the ulnar wrist articular capsule) could not restore DRUJ stability.^[41,46]^ Other researchers^[47,48]^ demonstrated that the application of soft tissue suture technology to suture the torn TFCC to the extensor carpi ulnaris tendon and ulnar articular capsule could treat TFCC damage.

Wrist joint arthroscope assessment and treatment of TFCC are essential for the operation and an important measure in preventing chronic pain in the wrist joint. Indeed, TFCC integrity should be thoroughly investigated under wrist joint arthroscopy. TFCC tissues should be retained even in cases of damage, and resection is not preferred because of its key role in wrist joint stability. The majority of TFCC damage is tears in the center, which can be treated by debridement under the wrist joint arthroscope. Specifically, a plasma knife or electric planer can remove the loosely ripped flap, trim it into a smooth and natural running state, and create a stable edge under stress, preventing joint interlocking due to torn flap confinement. Sutures should be placed under wrist joint arthroscopy for tears surrounding the TFCC with a blood supply.

The ability to treat patients with a distal radius fracture and DRUJ instability with simultaneous TFCC repair and internal fixation of the fracture has been debated. Mark et al^[49]^ proposed that anatomical reduction of the distal radius fracture should be considered first. The DRUJ of most patients could be restored to stability when they experienced DRUJ instability after open reduction and internal fixation of the distal radius fracture. After anatomical reduction, TFCC repair should be considered for patients with instability. According to Orbay,^[49]^ the distal fracture of the radius was not damaged in most cases of the distal interosseous membrane. The tension of the distal interosseous membrane returned with the reduction of the distal radius fracture, which could offer sufficient DRUJ stability for forearm rotation. Owing to the abundant blood supply at the ulnar TFCC in the late stage, the torn TFCC healed spontaneously and restored the stability of the DRUJ without repairing the TFCC. Other studies indicated that patients with DRUJ instability in many places should be evaluated for TFCC repair, and that TFCC damage associated with distal fracture of the radius was difficult to mend without surgical therapy.^[50,51]^

A previously reported repair was referred to in our case. Adams and Berger^[46,52]^ popularized a method that replicates both the orientation and function of palmar and dorsal distal radioulnar ligaments. In their reconstruction, a tendon graft was inserted during regeneration through the distal radius using a dorsaltovolar bone tunnel. The graft ends are then wrapped around the ulnar neck and joined together as a half-hitch after passing through an oblique tunnel in the distal ulna.

For reconstruction and repair in this case, the autologous ipsilateral palmaris longus tendon was used as the transplant.^[53]^ Contrary to Adams and Berger surgery, the TFCC was repaired using the palmar approach, and the distal radioulnar ligament was repaired by transplanting the palmaris longus tendon. After the operation, the patient returned to normal life and work, and grip strength recovered without wrist joint pain, wrist joint instability, or other complications.

Kakar et al^[54]^ classified various reconstruction methods to stabilize the DRUJ into 4 categories based on the following principles: (1) anatomical reconstruction of the distal radioulnar ligament,^[45,55]^ (2) contraction of the extensor retinaculum and articular capsule,^[56,57]^ (3) fixation of the suspensory tendon of the ulnar wrist,^[58,59]^ and (4) external distal radioulnar fixation.^[60,61]^ Reconstruction technologies based on tendon transplantation have steadily advanced over the past 30 year.^[45,53,62,63]^ According to Gutierrez et al, almaris longus tendon is transplanted to reconstruct the DRUJ ligament. During a 6-month follow-up after the operation, electron microscopy revealed that the transplanted palmaris longus tendon was revascularized and that its fiber structure was reconstructed, close to the normal ligament tissues.^[64]^ Biomechanical analysis by Scheker et al^[65]^ revealed that reconstruction of the dorsal radioulnar ligament by tendon transplantation was better than flexor carpi ulnaris reconstruction. Müller et al^[66]^ proposed the AO/ASIF classification system based on the severity of bone and joint injuries, which divided distal fractures of the radius into 3 categories: extra-articular fracture (A), simple or partial intra-articular fracture (B), and complex intra-articular fracture (C). Each type of fracture is divided into 3 subtypes based on the presence of comminution, impaction, metacarpal, dorsal, intra-articular, or metaphyseal comminution. Fernández^[67]^ then made further modifications and supplements the classification system to distinguish whether the fracture was stable or not, and complications were added to the classification. The system was made more useful by classifying pediatric fractures in a similar manner. A retrospective analysis of 55 cases of closed reduction and needle fixation for radial distal fractures of the C1 and C2 types was performed by Glickel.^[68]^ The results demonstrated that the patients made a good recovered well and that fracture recovery was equivalent to plate internal fixation. Additionally, it has been demonstrated that the fixation of distal fractures of the radius with Kirschner wires had the same effect as the plate for unstable extra-articular fractures or simple intra-articular fractures.^[69]^ Varitimidis et al^[70]^ observed that the fractured mass was reduced to the level of the articular surface and then fixed with Kirschner wires while being observed under a microscope and X-ray fluoroscopy, which was found to be more effective than X-ray fluoroscopy alone. Analysis of the morphological and biomechanical measurements of the interosseous ligaments of the DRUJ in this group revealed that the morphology and biomechanical features of each ligament were closely related to its physiological function to achieve anatomical restoration, which in turn reduced pain, increased grip strength, and improved function. It was necessary to restore a structure analogous to the TFCC or to repair the tension and integrity of the DRUJ ligament.^[71]^

## 5. Conclusion

Finite element analysis is one of the most critical tools in orthopedic biomechanics. It applies to both linear and nonlinear analysis, static and dynamic analysis, simple bone mode, and a complete model involving soft tissues such as muscles, ligaments, and vessels. This made the results more accurate and reliable. Therefore, finite element methods have significant implications for 3D printing, rapid rehabilitation, understanding the causes of fractures, improving artificial implants, and orthopedic surgery technology. Although finite element analysis can theoretically simulate any structure, it has limitations in the assumptions of material properties, boundary condition settings, and element number division. Therefore, the results from the finite element analysis are not entirely correct. Additionally, the resulting research objects were continuous with other tissues in the CT image processing. Therefore, the modeling process used manual repair. An anatomical atlas was used for manual assembly of the soft tissue and bone. These factors may affect the accuracy of the model, but they still provide reliable clinical recommendations. Future collaboration between researchers and professionals in imaging, kinematics, and computer science will be essential, as multidisciplinary cooperation is the best approach to fully exploit the potential of finite element analysis.

## Acknowledgments

We would like to thank the patients and their families for their assistance. We appreciate the contributions of all the patients, their families, investigators, and medical staff. We are grateful to all the authors.

## Author contributions

**Conceptualization:** Baiyang Zhang, Xilin Liu, Hongbin Sun.

**Data curation:** Baiyang Zhang, Xilin Liu.

**Funding acquisition:** Xilin Liu.

**Investigation:** Xilin Liu.

**Supervision:** Xilin Liu, Hongbin Sun.

**Writing – original draft:** Xilin Liu.

**Writing – review & editing:** Baiyang Zhang, Xilin Liu, Hongbin Sun.
